# Supplementary material for: An optimized LSTM network for improving arbitrage spread forecasting using ant colony cross-searching in the K-fold hyperparameter space
Source: PeerJ Comput Sci. 2024 Aug 9;10:e2215. doi: 10.7717/peerj-cs.2215 (PMC11639137; doi:10.7717/peerj-cs.2215)
Supplement: Supplemental Information 3 [file peerj-cs-10-2215-s003.zip › cec19/result.pdf]

Since the Modified Schwefel's Function of CEC19 has a negative optimization result, it is not applicable to the KCS algorithm. And we only selected the remaining nine functions for testing. The CEC19 test function is provided by the third-party library opfunu in Python. The dimensions of each function and search range are shown in Table 1. We tested three functions in total: ACO, KCS and Ant Colony Optimization Continuous (ACOR). Among them, ACO is the traditional ACO algorithm (heuristic functions for computing probability matrices are not included in this paper, as heuristic function design is often difficult for black-box models). ACOR is a variant of ACO provided by mealpy, a third-party library for Python. In addition, due to the large distribution of fitness, the Q value in the KCS algorithm is dynamically adjusted as the hyperparameter space converges. The number of populations for all algorithms is 50. In order to guarantee the reliability of the experimental results, each algorithm's search for the CEC19 function was performed 30 times independently. And we calculated the mean and standard deviation of its searched global optimal fitness, which are shown in Table 2.

Table 1. CEC2019 test functions.

No.	Functions	Dim	Search Range
1	Storn's Chebyshev Polynomial Fitting Problem	9	[-8192,8192]
2	Inverse Hilbert Matrix Problem	16	[-16384,16384]
3	Lennard-Jones Minimum Energy Cluster	18	[-4,4]
4	Rastrigin's Function	10	[-100,100]
5	Griewangk's Function	10	[-100,100]
6	Weierstrass Function	10	[-100,100]
7	Expanded Schaffer's F6 Function	10	[-100,100]
8	Happy Cat Function	10	[-100,100]
9	Ackley Function	10	[-100,100]

The N value in the table indicates how many times CEC19 was queried when the optimization algorithm first searched for best fitness. '*' indicates that the algorithm has reached the maximum number of iterations. At 1000 iterationss, both ACO and ACOR achieved minimum fitness on four functions and KCS achieved minimum fitness on only one function. With the maximum number of queries available for the CEC19 functions of 250,000, ACO achieves the minimum fitness on three functions, ACOR achieves the minimum fitness on all four functions and KCS achieves the minimum fitness on two functions. However, with an increase in the number of iterations, the maximum improvement of ACO in CEC19 functions is only 5.3%, except for F8. Additionally, the effect of individual functions becomes worse. The ACO algorithm's dependence on the number of populations is the reason for this situation. For both KCS and ACOR, the search results improve as the number of iterations increases. Finally, The poor performance of the KCS algorithm on the CEC19 test function can be attributed to its focus on global search with poor local exploration.

Table 2. Avg and Std results using CEC 2019 test functions.

CEC19 function	global best fitness	1000 iterations			5000 iterations		10000 iterations
		ACO	KCS	ACOR	ACO	KCS	ACOR
F1	Avg	13207022	**2828146**	16479268	12513435	2716626	**1056901**
	std	12988350	3780442	6676532	10168788	2787377	1294822
	N	6200	31293*	18051	4150	141626	241391
F2	Avg	**527**	827	1133	679	**521**	605
	std	277.86	477.75	187.54	401.42	160.58	126.04
	N	6200	33952*	13476	4300	156113*	147216
F3	Avg	10.72	13.50	**10.60**	10.45	12.92	**5.27**
	std	0.774	0.377	0.759	1.077	0.570	0.427
	N	3250	31407*	16551	1760	116616	177651
F4	Avg	122.62	111.54	**39.59**	209.68	**19.24**	25.02
	std	157.27	115.28	3.73	350.70	14.84	3.35
	N	5250	31250*	16076	3150	104603	152892
F5	Avg	**1.11**	2.26	1.57	**1.11**	2.14	1.45
	std	0.055	0.142	0.081	0.057	0.105	0.080
	N	5250	29660*	12501	3150	126484	100014
F6	Avg	**3.65**	20.09	10.85	**3.73**	7.73	9.66
	std	1.198	2.514	0.895	1.037	2.188	0.50
	N	5250	29139*	10826	3150	123547	116604
F7	Avg	1.001	1.106	**1.000**	1.000	1.068	**1**
	std	0.0025	0.0369	3.73e-16	0.0012	0.0357	0
	N	5250	29512*	24826	3150	127795	25059
F8	Avg	6.53	2.94	**1.30**	4.45	1.75	**1.15**
	std	9.527	1.595	0.052	4.325	1.216	2.79e-02
	N	5200	31384*	19451	3100	103662	179301
F9	Avg	**20.95**	22.75	21.45	**20.89**	22.47	21.30
	std	0.234	0.166	0.087	0.416	0.291	0.051
	N	5250	28944*	13201	3150	133169	108366
